# Combined transcriptome and metabolome analysis reveals breed-specific regulatory mechanisms in Dorper and Tan sheep

**DOI:** 10.1186/s12864-023-09870-9

**Published:** 2024-01-17

**Authors:** Yuhao Ma, Ganxian Cai, Jianfei Chen, Xue Yang, Guoying Hua, Deping Han, Xinhai Li, Dengzhen Feng, Xuemei Deng

**Affiliations:** 1 Key Laboratory of Animal Genetics, Breeding, and Reproduction of the Ministry of Agriculture and Beijing Key Laboratory of Animal Genetic Improvement, China Agricultural University, Beijing, 100193 China; 2 Department of Animal Science and college of Agriculture, Ningxia University, Yinchuan, 750021 China

**Keywords:** Dorper sheep, Tan sheep, Transcriptome, Metabolome, Host metabolic network

## Abstract

**Background:**

Dorper and Tan sheep are renowned for their rapid growth and exceptional meat quality, respectively. Previous research has provided evidence of the impact of gut microbiota on breed characteristics. The precise correlation between the gastrointestinal tract and peripheral organs in each breed is still unclear. Investigating the metabolic network of the intestinal organ has the potential to improve animal growth performance and enhance economic benefits through the regulation of intestinal metabolites.

**Results:**

In this study, we identified the growth advantage of Dorper sheep and the high fat content of Tan sheep. A transcriptome study of the brain, liver, skeletal muscle, and intestinal tissues of both breeds revealed 3,750 differentially expressed genes (DEGs). The genes *PPARGC1A*, *LPL*, and *PHGDH* were found to be highly expressed in Doper, resulting in the up-regulation of pathways related to lipid oxidation, glycerophospholipid metabolism, and amino acid anabolism. Tan sheep highly express the *BSEP*, *LDLR*, and *ACHE* genes, which up-regulate the pathways involved in bile transport and cholesterol homeostasis. Hindgut content analysis identified 200 differentially accumulated metabolites (DAMs). Purines, pyrimidines, bile acids, and fatty acid substances were more abundant in Dorper sheep. Based on combined gene and metabolite analyses, we have identified glycine, serine, and threonine metabolism, tryptophan metabolism, bile secretion, cholesterol metabolism, and neuroactive ligand-receptor interaction as key factors contributing to the differences among the breeds.

**Conclusions:**

This study indicates that different breeds of sheep exhibit unique breed characteristics through various physiological regulatory methods. Dorper sheep upregulate metabolic signals related to glycine, serine, and threonine, resulting in an increase in purine and pyrimidine substances. This, in turn, promotes the synthesis of amino acids and facilitates body development, resulting in a faster rate of weight gain. Tan sheep accelerate bile transport, reduce bile accumulation in the intestine, and upregulate cholesterol homeostasis signals in skeletal muscles. This promotes the accumulation of peripheral and intramuscular fat, resulting in improved meat quality. This work adopts a joint analysis method of multi-tissue transcriptome and gut metabolome, providing a successful case for analyzing the mechanisms underlying the formation of various traits.

**Supplementary Information:**

The online version contains supplementary material available at 10.1186/s12864-023-09870-9.

## Background

A variety of factors, including gene expression, metabolism, environment, feed, and microorganisms, influence animal growth and development [1]. These factors interact and contribute to the breed-specific characteristics [2, 3]. Metabolism plays a pivotal role in shaping development, as it converts food into energy [4]. The close relationship between the host's gut and peripheral organs plays a pivotal role in the host's development [5, 6]. Research has shown that the intestines break down food into various substances that travel through the bloodstream, thereby affecting the peripheral organs. These organs, in turn, regulate the function of the intestine [7]. Several studies have established a link between microbial dysbiosis and acute or chronic diseases. Pro-inflammatory microbiota generate metabolites that travel through different intestinal organ axes and reach extraintestinal organs, contributing to disease [8]. Individuals with obesity and non-alcoholic fatty liver disease exhibit alterations in intestinal metabolites due to metabolic disorders [9]. Therefore, exploring the interaction between the intestines and peripheral organs offers a promising avenue for investigating growth traits and metabolic variations. Recent studies have emphasized the significance of the gut-brain axis [10], gut-liver axis [11], and gut-muscle axis [12] as promising areas of investigation.

Previous studies have investigated the impact of various factors, including altitude [13], feeding habits [14], and geographic location, on gut microbes and metabolites [15]. However, there is a lack of a comprehensive systems perspective for understanding animal traits. While some studies have integrated transcriptomic and metabolomic approaches, they have focused on single target features [16, 17]. In our previous study, we identified the participation of *Lactobacillus* and *Phascolarctobacterium* in nucleotide metabolic processes in the intestine of Dorper sheep. Additionally, we observed increased lipid accumulation in Tan sheep [18]. Nevertheless, the influence of metabolite-organ tissue interactions on lipid metabolism and growth characteristics remains unclear. Establishing the sequence of intestinal metabolites and investigating the metabolic network between the intestines and other organs are essential.

This study aims to investigate the metabolic relationships between the intestines and peripheral organs in Dorper and Tan sheep. Tan sheep is a locally recognized breed known for its exceptional meat quality, and it currently dominates the lamb market in China [19]. Meanwhile, the Dorper is a widely used meat sheep breed known for its fast growth and high meat production [20]. For this study, we utilized phenotypic analysis, tissue section observation, metabolomics, and transcriptomics to identify species-specific traits and systematically construct host metabolic networks. The findings from this study are expected to establish a foundation for a systematic understanding of animal metabolism, ultimately leading to improvements in animal health and productivity.

## Results

### Serum lipid metabolism, inflammatory parameters, and sections of the intestine and liver

**Fig. 1:**
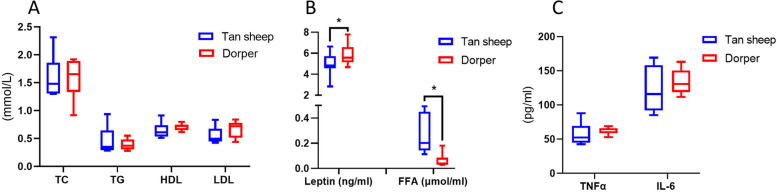
Parameters of Dorper and Tan sheep serum samples at D240. **A** Lipid metabolism parameters. **B** Glucose parameters. **C** Concentration of inflammatory cytokines. Data are presented as box plots. *P < 0.05

**Fig. 2:**
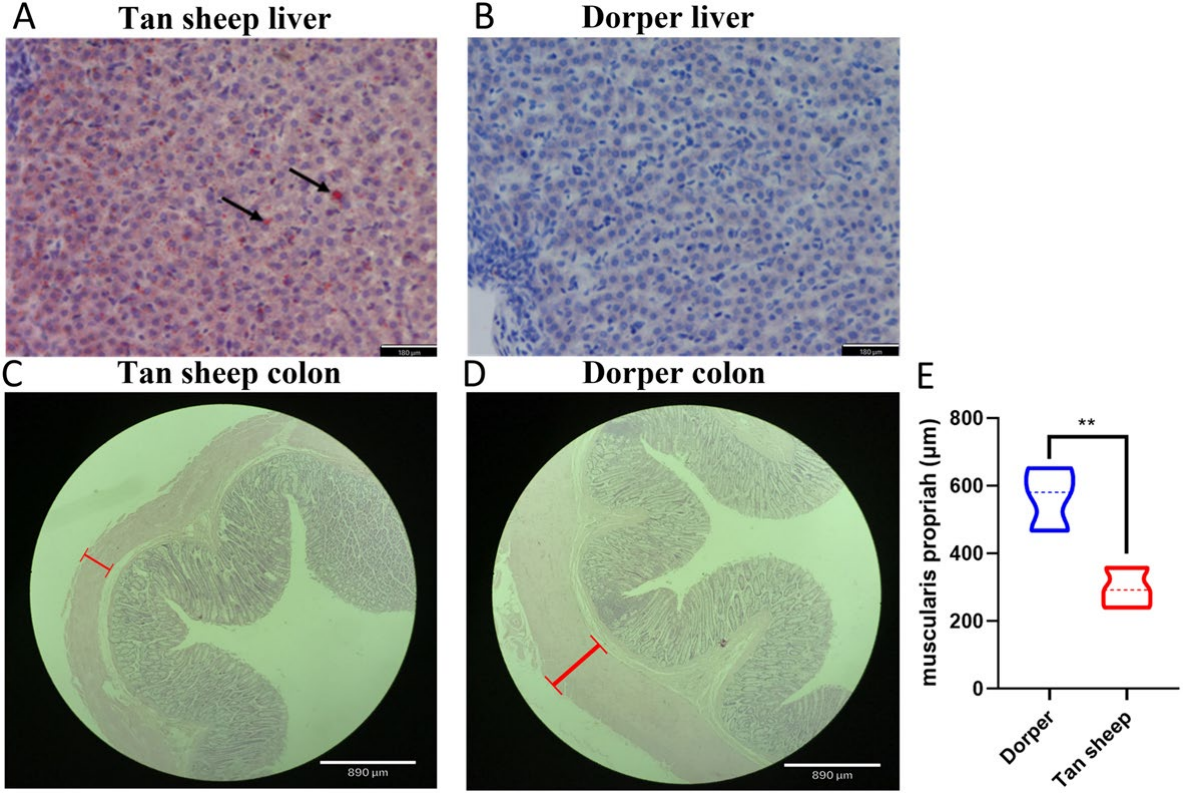
Histological observations and statistics of Dorper and Tan sheep at D240. **A**, **B** Oil red O staining results of liver tissue from Tan sheep and Duper sheep. Arrows point to lipid droplets in the liver. **C**, **D** Results of hematoxylin–eosin staining of colon tissues from Tan sheep and Dorper sheep. Muscularis propria is marked using red line segments. **E** Statistics on the thickness of intestinal muscularis propria of Dorper sheep and Tan sheep. Data are presented as violin plots. *n* = 5 for each group. **P < 0.01

In this study, we measured levels of lipid metabolism parameters in Dorper and Tan sheep at D240 to evaluate the breed characteristics between the two groups. We observed that plasma triglyceride (TG), total cholesterol (TC), low-density lipoprotein (LDL), and high-density lipoprotein (HDL) levels did not exhibit significant differences between the two groups. However, TG and TC concentrations were higher in Tan sheep. In addition, the Dorper sheep had a significantly lower free fatty acid content compared to the Tan sheep, while the leptin concentration was elevated (*P* < 0.05) (Fig. 1A and B). Since perturbation of lipid metabolic homeostasis has been linked to the pro-inflammatory response, we also examined the levels of the inflammatory factors IL-6 and TNF-α (Fig. 1C). Our results showed that the concentrations of IL-6 and TNF-α were higher in Dorper sheep compared to Tan sheep, although the difference was not statistically significant. We compared liver lipid accumulation between Dorper and Tan sheep. Oil Red O staining showed the accumulation of small lipid droplets in the liver of Tan sheep (Fig. 2A). In contrast, the liver tissue of Dorper showed virtually no aggregation of lipid droplets (Fig. 2B). Additionally, histological analysis of colon slices revealed that Dorper sheep had more villi compared to other breeds. This was attributed to the deepening of the intestinal plicae circulares, as shown in Fig. 2C, D. Statistical analysis of the muscularis propria thickness of the colon demonstrated that the muscularis propria was thicker and more developed in Dorper sheep (Fig. 2E).

### Sequencing and quality control of transcriptome data

**Fig. 3:**
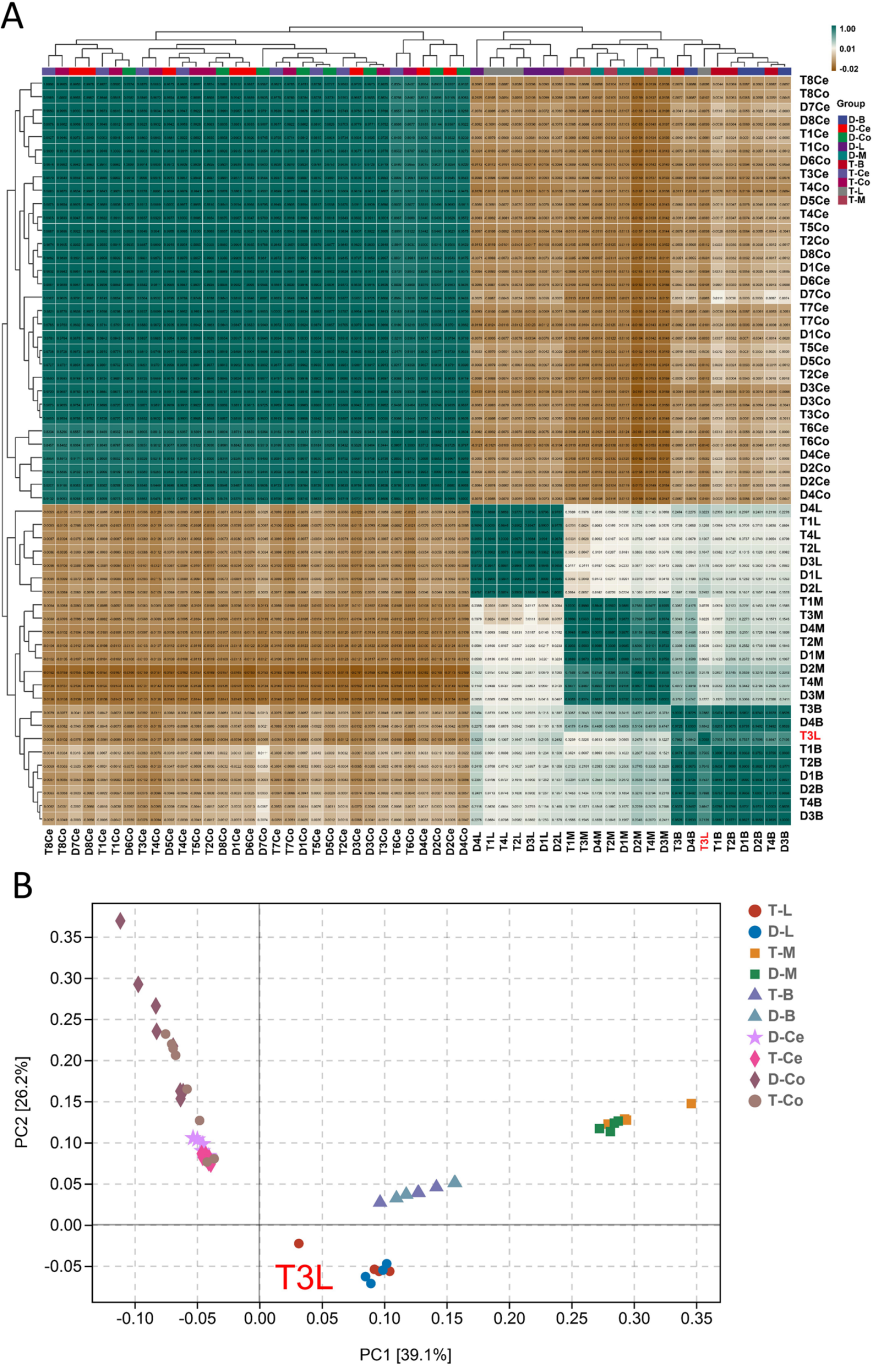
Gene expression analysis. **A** Cross-correlation coefficient plot between samples. **B** Principal coordinate analysis (PCoA) based on all samples. D, T indicate Dorper sheep and Tan sheep. B, L, and M indicate brain, liver, and muscle tissue, respectively. Number indicates the number of the individual

Sequencing yielded a total of 1,968,595,584 raw reads for the forty samples. Following quality control, we generated 1,843,580,854 clean reads, with a Q30 base percentage exceeding 94.08%. The percentage of clean reads mapped to the sheep reference genome ranged from 94.00% to 98.00%. Approximately 92% of the pure reads were uniquely mapped and used for subsequent analysis (refer to Supplemental Table S1). Pearson correlation analysis showed that brain, liver, and muscle tissues clustered separately, and the T3L samples were significantly distinct from liver tissues. This result was also verified through Principal Component Analysis (PCA). PCA by linear transformation revealed that PC1 and PC2 explained 47.1% and 22.2% of the variance, respectively. T3L samples were excluded from subsequent analysis due to significant deviations in gene expression (Fig. 3).

### Differential expression gene analysis and validation of DEGs using qRT-PCR

**Fig. 4:**
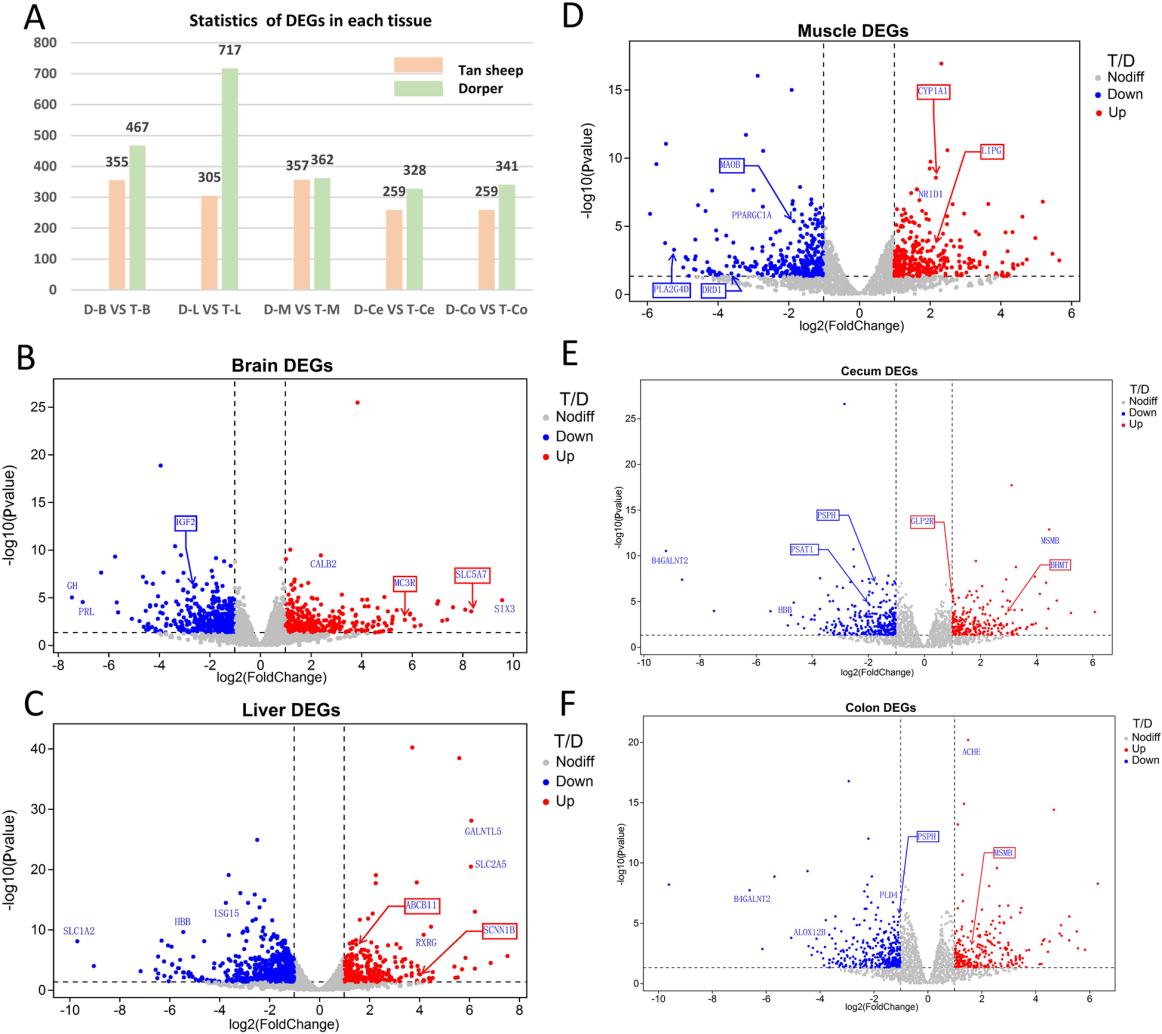
DEG expression analysis. **A** The number of DEGs in each tissue. **B**-**F** Volcano plot displaying DEGs in the brain, skeletal muscle, liver, cecum and colon

**Fig. 5:**
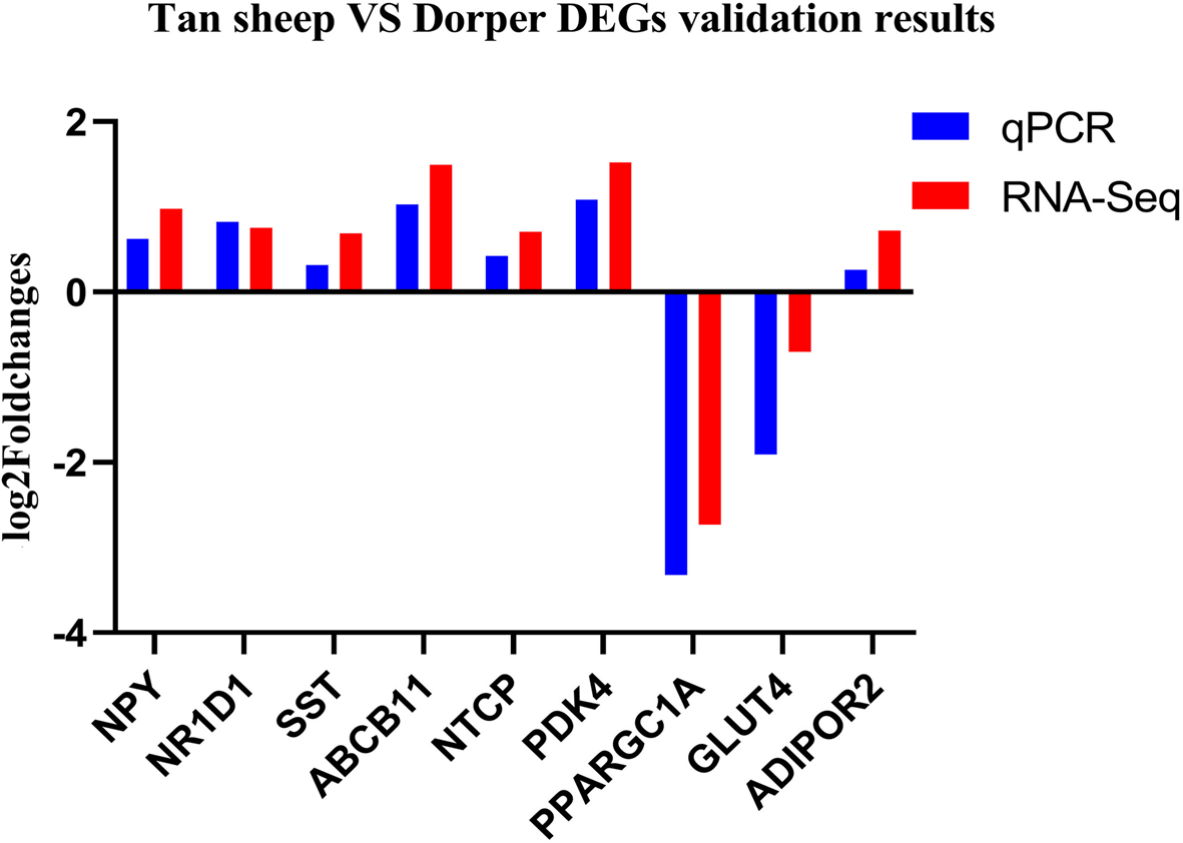
qRT-PCR validation of DEGs. log_2_Foldchanges > 0 indicated up-regulated gene expression in Tan sheep, and log_2_Foldchanges < 0 indicated up-regulated gene expression in Dorper sheep

The liver had the highest number of differentially expressed genes (DEGs), with a total of 1,022 DEGs identified (Fig. 4A, Supplemental Table S2). Volcano plots were generated to visualize the fold differences in gene expression and their significance in the brain, liver, skeletal muscle, cecum, and colon, respectively (Fig. 4B-F). Key genes with significant fold changes and *P-values* were highlighted, including *GH, PRL, ABCB11, PPARGC1A, ACHE, PSPH,* and other genes. We confirmed the RNA-Seq results by conducting qRT-PCR for nine DEGs in various tissues. Log2 fold changes from qRT-PCR were compared to the results of the RNA-Seq expression analysis. The results showed that the expression patterns of the nine DEGs were consistent with the results of RNA-seq analysis, further demonstrating the reliability of the transcriptome data (Fig. 5).

### Functional enrichment of differentially expressed genes

**Fig. 6:**
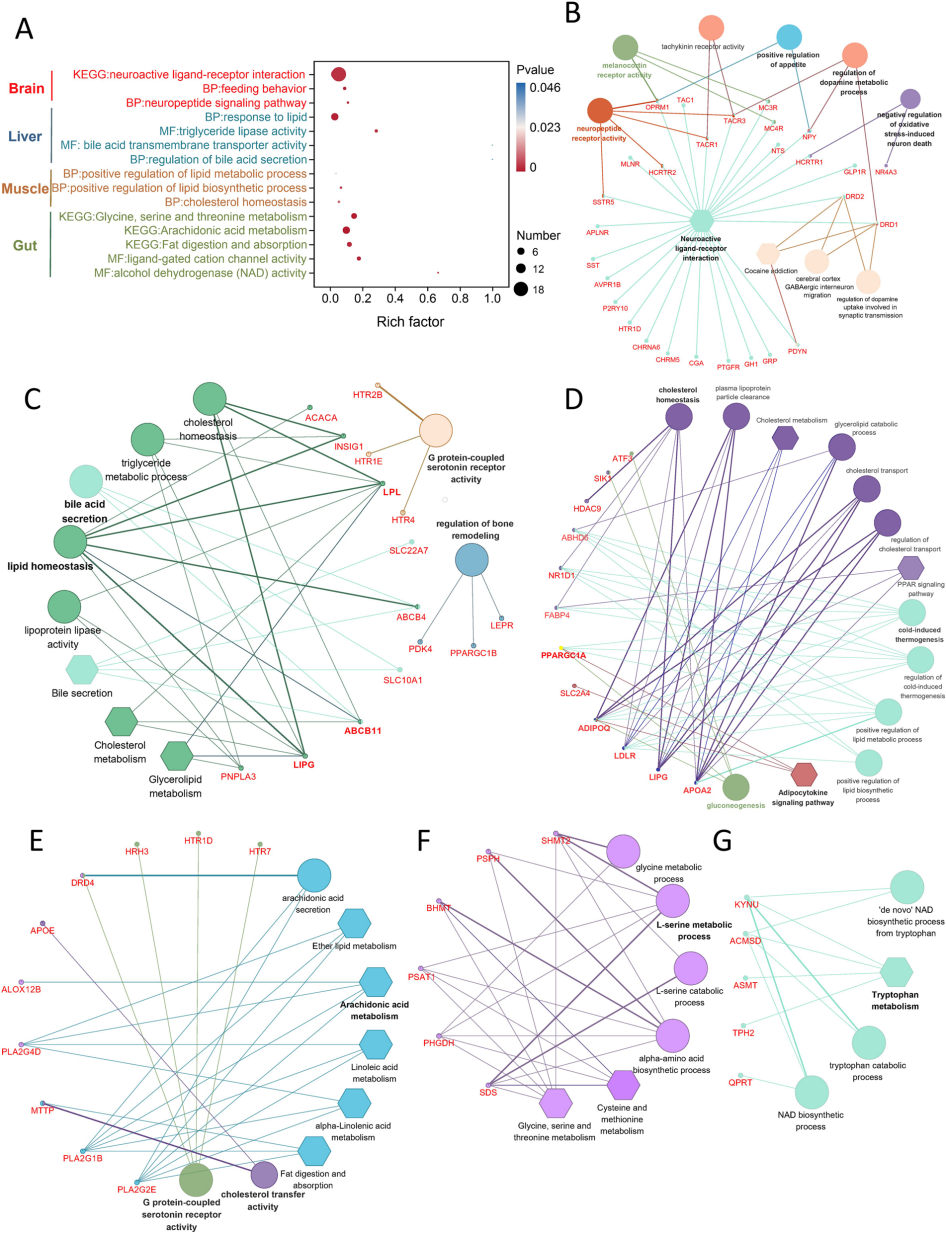
Functional annotation analysis of DEGs. **A** Enrichment results of key metabolic pathways for each group. **B**-**D** Gene network maps of brain, liver, and skeletal muscle groups realized by ClueGO. **E**–**G** Gene network maps of intestine groups realized by ClueGO. Edge thickness indicates the association strength while node size corresponds to the statistical significance for each term. Biological processes are represented by circles and KEEG pathways are represented by hexagons

GO and KEGG enrichment analyses were performed to investigate the biological functions involved in each tissue. Notably, immune-related genes were identified in both brain and liver tissues. To effectively demonstrate the metabolic processes related to animal characteristics, bubble plots were used to visualize the enrichment results for each tissue (Fig. 6A, Supplemental Table S3). The genes that were enriched in each tissue were associated with neural signals that regulate feeding behavior in the brain, bile acid and lipid metabolism in the liver, and lipid metabolism in skeletal muscle tissue (Fig. 6B-D). Three types of signals were enriched in intestinal tissues, including lipid metabolism, glycine/serine metabolism, and tryptophan metabolism (Fig. 6E-G). To further analyze gene information, we focused on the expression functions of key genes in these pathways. ClueGO analysis identified hub genes involved in metabolism, such as *MC4R, NPY, ABCB11, LIPG, PSPH,* and *PLA2G1B*.

### Profiles of the intestinal metabolome

**Fig. 7:**
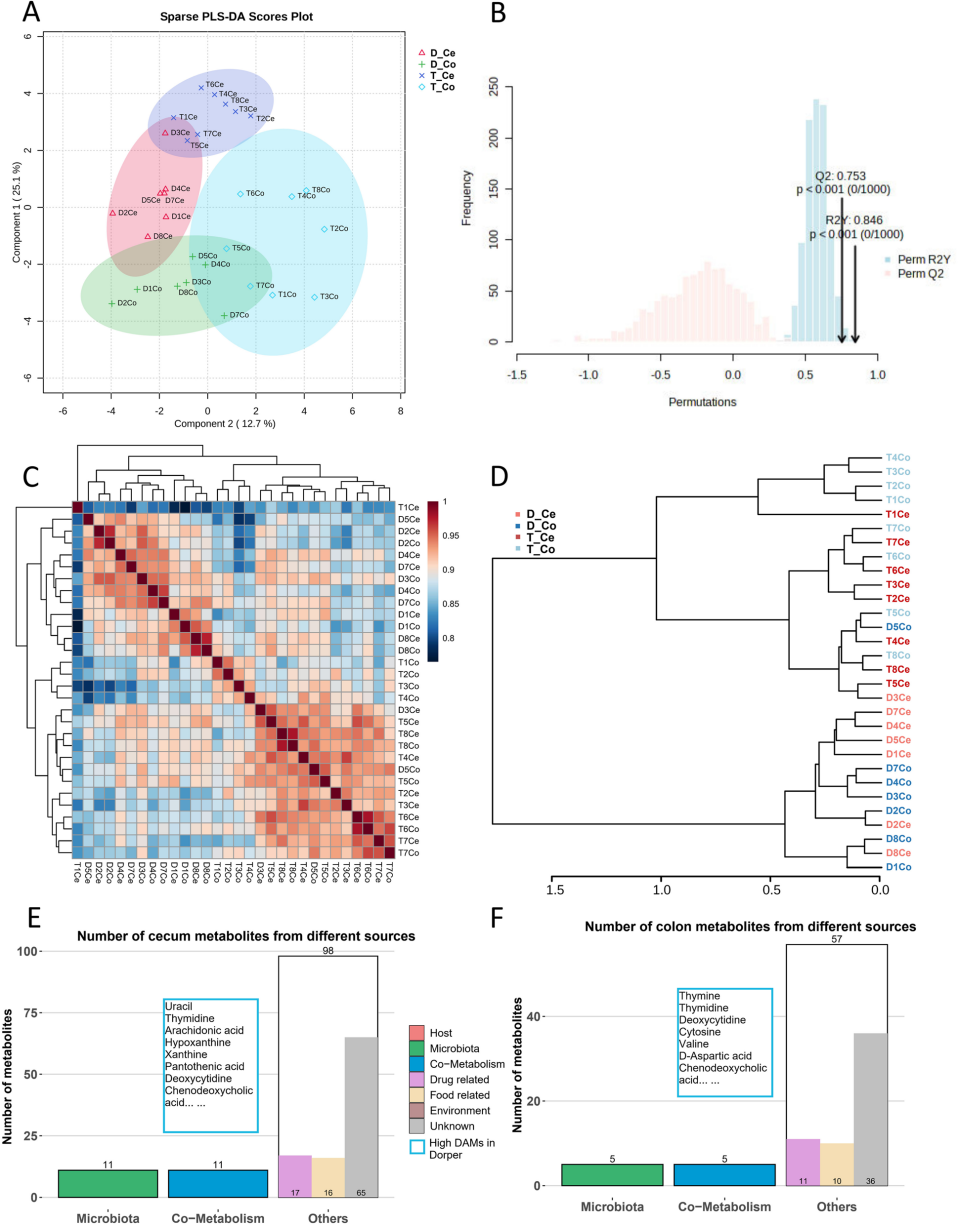
Profiles of Metabolome. **A** The sPLS-DA 2D score plot with 95% confidence regions. **B** Model validation permutation test for OPLS-DA. **C** Heat map of correlation between samples. **D** Hierarchical clustering dendrogram between samples. **E**, **F** Statistical analysis of source classification of cecum and colon metabolites

The PLS-DA analysis reveals a significant difference in sample clustering among the groups. Metabolites from different species were observed to fall within the 95% confidence interval (Fig. 7A). The established models R2 and Q2 were both greater than 0.5, as confirmed by the permutation test of orthogonal PLS-DA. This demonstrates that the models were stable, reliable, and suitable for comparing the differences between the two respective groups (Fig. 7B, Supplemental Figure S1). Pearson's correlation was used to calculate sample correlations, and it was observed that the specificity of intestinal tissue resulted in correlations greater than 0.7 for each sample. Moreover, higher correlations were observed for adjacent intestinal segments among individuals (Fig. 7C). The hierarchical clustering dendrogram, using the Euclidean distance measurement and Ward's clustering algorithm, revealed distinct clusters of breeds. There was no discernible clustering based on gut site or gender factors (Fig. 7D). Furthermore, using VIP > 1 and* P* < 0.05 as screening criteria, 130 and 70 differential metabolites were identified in the cecum and colon groups, respectively. The origins of microbial metabolites, host metabolites, and co-metabolism were differentiated (Fig. 7E, F, Supplemental Table S4).

### Functional enrichment of differentially accumulated metabolites

**Fig. 8:**
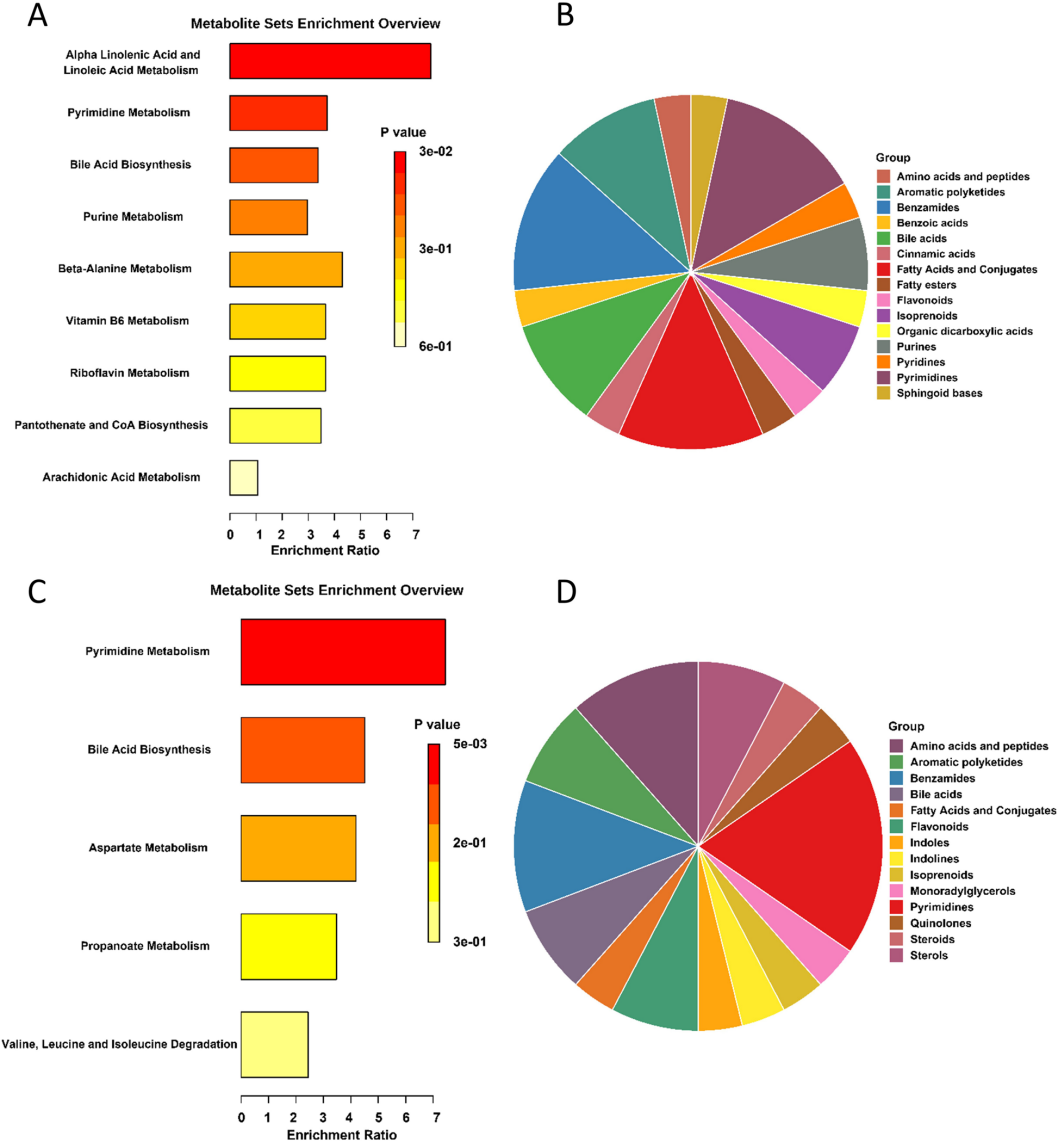
Functional enrichment of differentially accumulated metabolites. **A**, **C** Metabolic pathway analysis of cecum and colon metabolites based on the SMPDB database. **B**, **D** Classification of cecum and colon metabolites based on main-class chemical structures

Figure 8 displays the annotated results of the SMPDB pathway for differential abundance analysis. The cecum exhibited significant enrichment in the metabolism of alpha-linolenic acid and linoleic acid, nucleotides, bile acids, and amino acids. Similar metabolic processes were also enriched in the colon. These metabolites are primarily involved in bile acid and nucleotide metabolism. The analysis of cecum metabolites revealed that the main substances were fatty acids and their derivatives, benzamides, and pyrimidines. In the colon, the predominant substances belonged to the classes of pyrimidines, benzamides, amino acids, and peptides.

### Synergistic effects of differentially expressed genes (DEGs) and differentially abundant metabolites (DAMs)

**Fig. 9:**
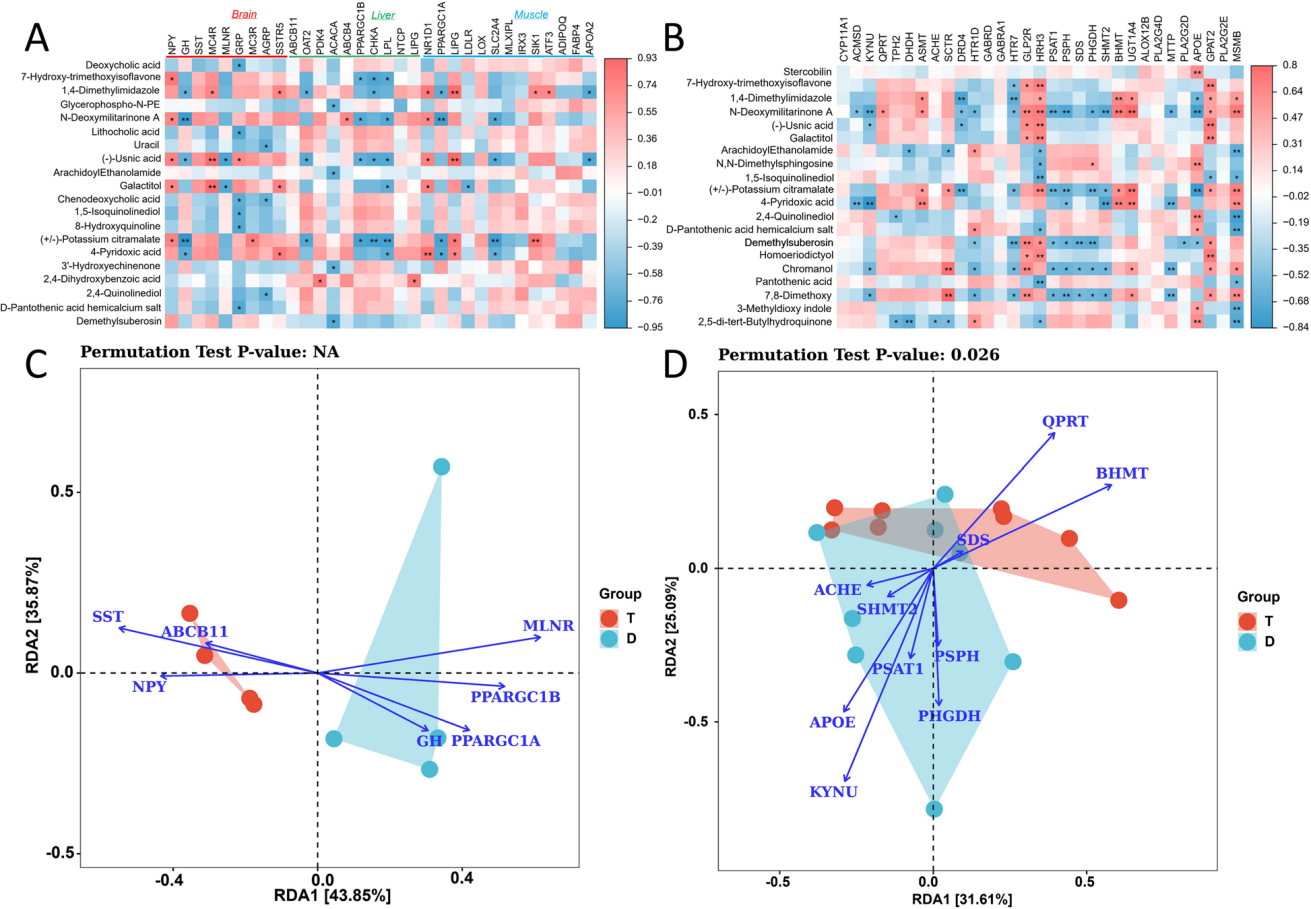
Correlation analysis. **A** Spearman's relationship between DAMs and peripheral organ hubs DEGs. **B** Spearman's relationship between DAMs and intestinal hubs DEGs. **C** Redundancy analysis between peripheral tissue genes and DAMs. **D** Redundancy analysis between intestinal hubs genes and DAMs

The Spearman correlation coefficient model was used to further analyze the correlation between candidate genes and key metabolites. We identified the relationships between the top 20 DAMs and hub DEGs, uncovering significant correlations between ( ±)-potassium citramalate and (-)-usnic acid with the expression of multiple genes in peripheral organs. Conversely, N-Deoxymilitarinone A and demethylsuberosin showed a significant correlation with the expression of multiple genes in intestinal tissues (Fig. 9A, B). Subsequently, RDA was used to determine the relationship between hub genes and metabolites. The results showed that the eigenvalues of the first axis of intestinal tissue were 0.316, and for the second axis, they were 0.250. *QPRT* and *BHMT* had the most significant impact on the metabolic fraction of Tan sheep, while *PSPH*, *KYNU*, and *APOE* had a greater effect on the metabolic fraction of Dorper (*P* < 0.05). In peripheral tissues, the first axis explains 43.8% of the data characteristics, while the second axis explains 35.9%. The metabolic fraction of Dorper sheep was primarily influenced by *MLNR*, *PPARGC1B*, and *PPARGC1A*, while *SST* and *NPY* had the main impact on the metabolic fractions of Tan sheep (Fig. 9C and D).

**Fig. 10:**
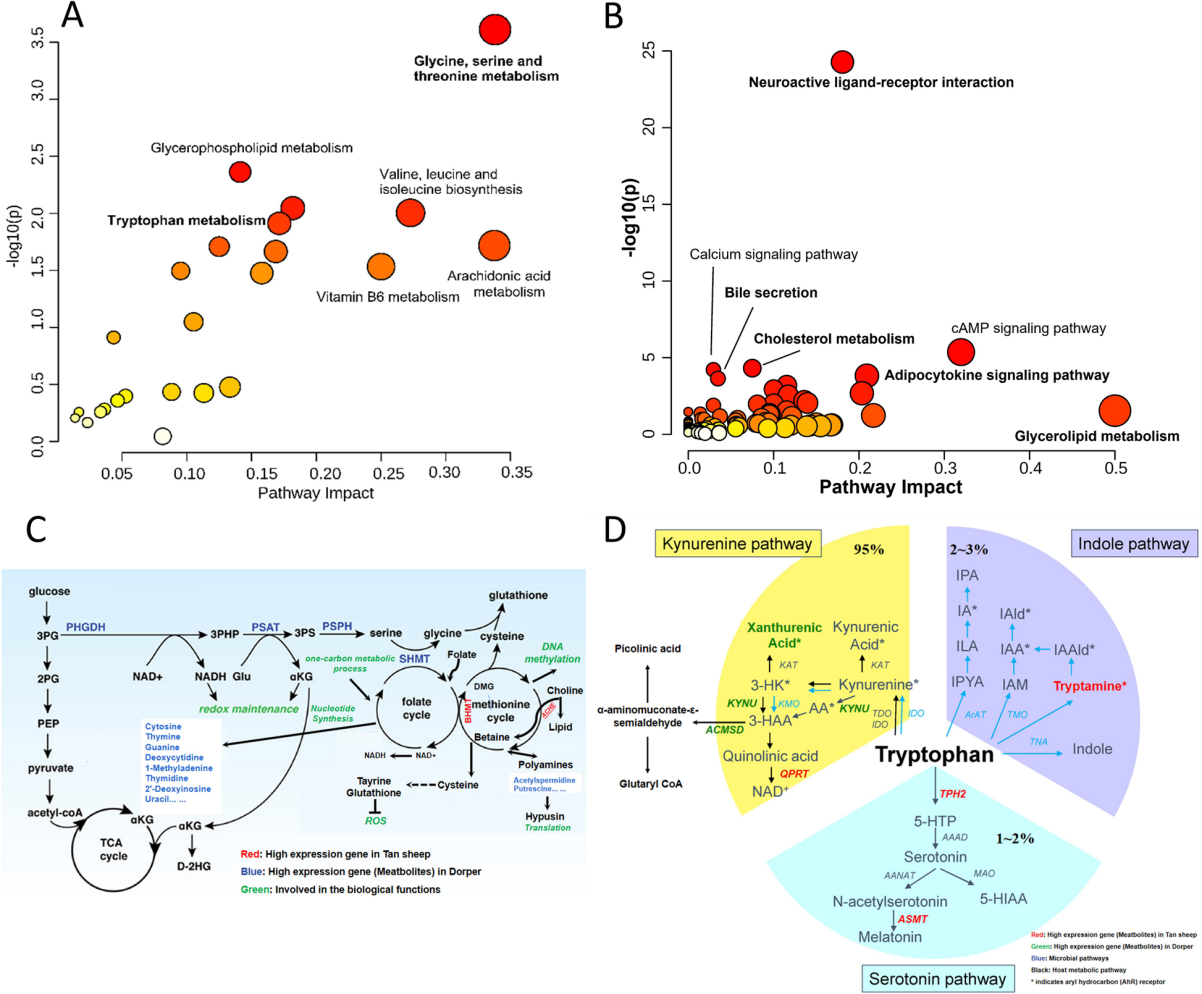
Function analysis of intestinal DEGs and DAMs. **A** Joint-pathway analysis between the metabolites and intestinal gene. **B** Joint-pathway analysis between the metabolites and peripheral tissues gene. **C** DEGs and DAMs in glycine, serine and threonine Metabolism. **D** DEGs and DAMs in tryptophan Metabolism

The analysis of shared metabolic pathways revealed a relationship between genes and metabolites. Significantly enriched processes between intestinal tissues and metabolites included glycine, serine, and threonine metabolism, glycerophospholipid metabolism, and tryptophan metabolism (Fig. 10A). In peripheral tissues, the joint pathway analysis of metabolites and genes led to the enrichment of pathways such as neuroactive ligand-receptor interaction, bile secretion, and cholesterol homeostasis (Fig. 10B). Among them, we focused on visualizing biological pathways involving several genes and metabolites in the intestine. High-expression genes, such as *PHGDH*, *PSPH*, and *SHMT*, are expressed at higher levels in Dorper sheep. These genes have an impact on nucleotide production through glycine biosynthesis and one-carbon metabolism. Interestingly, Dorper exhibited a higher abundance of nucleotide substrates. The high-expression genes *KYNU* and *ACMSD* increase the production of xanthine and quinoline, a process that seems to be more pronounced in the Dorper breed. As shown in Fig. 10C and D (Image modified from Richa Rathore [21] and Chien-Ning Hsu [22]), we have mapped the information of the genes and metabolites onto the metabolic network.

### Construction of metabolic network profiles of host intestine and peripheral tissues

**Fig. 11:**
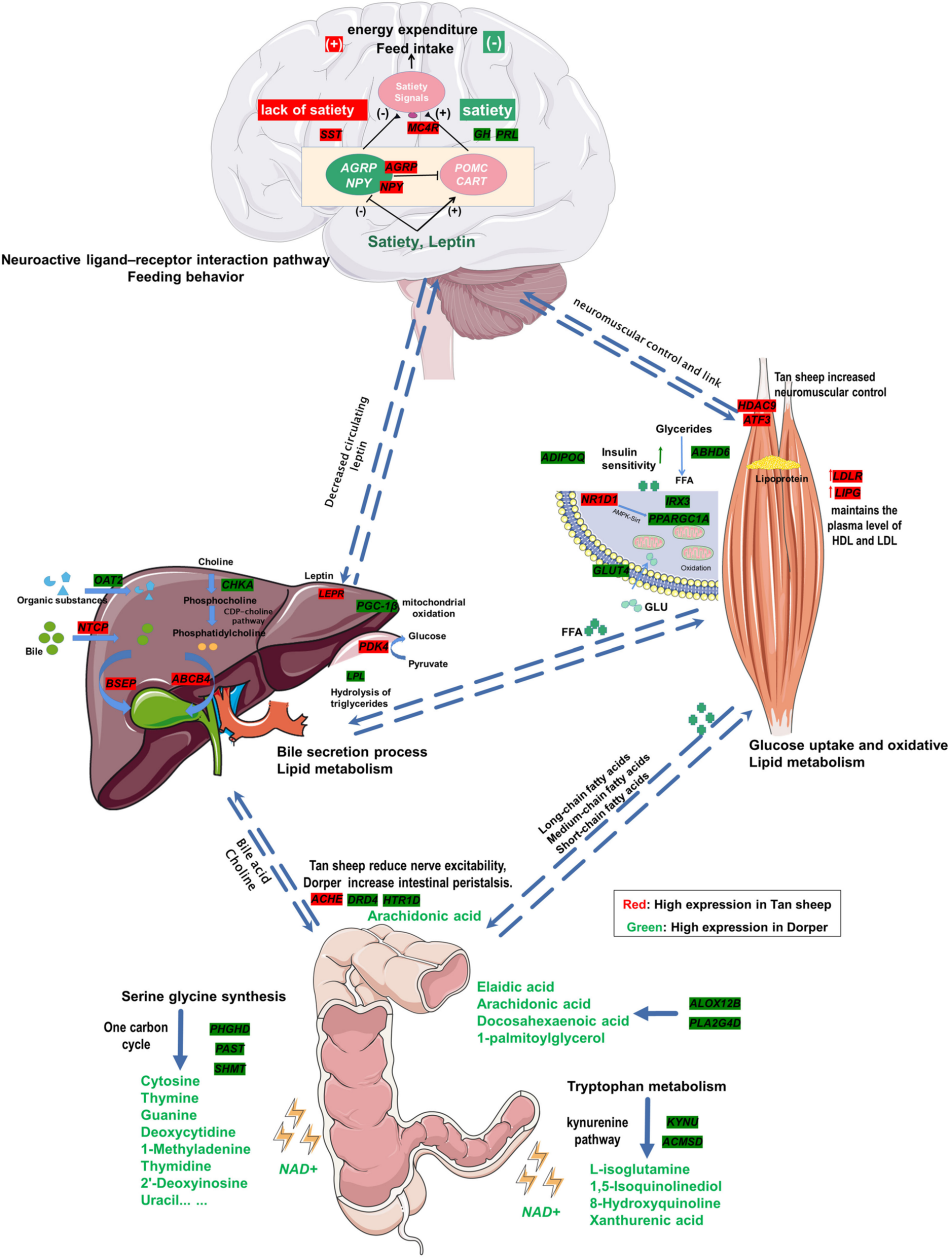
Schematic model showing differences in the metabolic network of Dorper and Tan sheep. Dark green was the high expression gene of Dorper, and dark red was the high expression gene of Tan sheep

Employing a combination of transcriptomic and metabolomic data, we constructed a pathway map that elucidates the impact of Dorper and Tan sheep on enterohepatic circulation, fatty acid oxidation, and feeding pathways (Fig. 11). We identified several genes that were highly expressed in Tan sheep. These genes include *NTCP*, *BSEP*, and *ABCB4* in the liver, *SST*, *NPY*, and *AGRP* in the brain, *LIPG*, *LDLR* in skeletal muscle, and *ACHE* in the intestine. Furthermore, we identified DEGs related to lipid metabolism, including *PPARGC1A*, *GLUT4*, *LPL*, and *ADIPOQ*. The study found that Tan sheep had a reduced accumulation of bile in the cecum, which was attributed to an intensified process of liver bile acid transport. Conversely, in Dorper sheep, hindgut amino acid metabolism increases the production of nucleic acid substrates, which contributes to the development of the gut muscularis propria and the body. Furthermore, Dorper sheep exhibited increased lipid oxidation and reduced fat accumulation. Additionally, the study proposed that gut-brain interactions play a pivotal role in stimulating intestinal signaling and peristalsis in Dorper sheep.

## Discussion

Animal development is a complex process involving the coordinated organization of multiple organs, which is influenced by both genetic programming and environmental factors [23]. Various organs employ different types of information to regulate specific traits. For instance, liver growth is regulated by the flow of bile acids in the intestine, while signals such as *FXR* and *TGR5* control compensatory circulation in the liver [24]. Myocytes throughout the body produce and release inhibitors of muscle growth, which can negatively regulate the growth of skeletal muscle [25]. The integrative analysis of multiple organs can efficiently extract host genetic information and provide innovative strategies for conducting comprehensive studies.

Gene expression of liver bile transport has a direct impact on the amount of bile acids in the intestine, which plays a crucial role in cholesterol homeostasis [26] and the regulation of lipid and glucose metabolism [27, 28]. The gut-liver axis is tightly connected through bile acids, which facilitate the digestion of dietary fats and oils [29]. Our metabolomics analysis revealed higher levels of bile acids in the intestines of Dorper sheep, including chenodeoxycholic acid and deoxycholic acid. This suggests a more intense process of catabolism of emulsified cholesterol in the hindgut. This process can supply energy substances, such as fatty acids, to other tissues in the organism [30]. The *BSEP* gene plays a crucial role in maintaining enterohepatic circulation and bile flow. It functions by facilitating the secretion of bile salts from hepatocytes into the bile [31]. Likewise, *NTCP* is involved in the sodium-dependent uptake of bile acids and, therefore, plays a role in the enterohepatic circulation [32]. *ABCB4* transfers phosphatidylcholine from the inner lobule of the hepatocyte canalicular membrane into bile [33]. These genes are up-regulated in the liver of Tan sheep, promoting increased resistance to liver lipid accumulation by facilitating greater bile flow and secretion of bile salts, phosphatidylcholine, and cholesterol [34]. *OAT2* and *CHKA* are also involved in mediating the transport of other substrates and the synthesis of phosphorylcholine [35]. Previous studies have demonstrated that bile acids play a role in mucosal protection and barrier functions, although they do not affect feed intake or body weight changes [36]. Studies have also found that knockdown of the *MSTN* gene in cattle enhances bile acid biosynthesis and regulates the process of bile acid metabolism, possibly through the intestinal muscle axis [37]. Our results confirm that enhanced bile acid transport decreases the quantity of bile acid in the hindgut, potentially resulting in the buildup of fat in the liver and skeletal muscle.

Integrating DAMs and DEGs revealed that the serine and glycine synthesis processes were more active in Dorper sheep, while a distinct tryptophan metabolic pathway was observed in Tan sheep. Dorper sheep, characterized by rapid growth and high muscle content, exhibited more robust development of the intestinal muscularis propria. Muscle tissue development requires nucleic acid substrates and protein synthesis, both of which are supported by the serine biosynthetic pathway. Serine serves as a precursor to several amino acids, and its biosynthetic pathway is a crucial step in glucose conversion [38, 39]. Furthermore, serine can be converted to glycine, providing carbon units for one-carbon metabolism. One-carbon metabolism is a series of reactions that supplies methyl groups for various biological methylation reactions, including nucleotide metabolism, purine and pyrimidine synthesis, and amino acid metabolism [39]. High expression of the *PHGDH*, *PSAT1*, and *SHMT2* genes promotes serine synthesis in Dorper sheep, resulting in increased purine production, TCA cycle activity, and NADH production [40]. This ultimately leads to improved development of the gut muscularis propria in Dorper sheep. While serine biosynthesis and one-carbon metabolism have been extensively studied in cancer research and gestational development [41, 42], their combined metabolome in animal development has not been extensively reported.

Tryptophan is a biosynthetic precursor of a large number of microbial and host metabolites, including three metabolic pathways [43]. In the kynurenine pathway, the enzyme encoded by *ACMSD*, *KYNU*, is highly expressed in Dorper sheep. Intermediate metabolites in this pathway include xanthine, which ultimately leads to the production of quinolinic acid. This observation is consistent with the metabolomic results, which showed higher abundance of xanthine and quinoline in the gut of Dorper sheep. These metabolites contribute to the generation of NADH, which transports electrons for the organism's energy metabolism.

Our analysis of differentially expressed genes (DEGs) related to lipid metabolism synthesis indicates that Tan sheep tend to accumulate more fat, while Dorper sheep exhibit a higher rate of fat oxidation. In the liver, overexpression of *PPARGC1B* has been linked to increased mitochondrial respiration and energy expenditure in mice, resulting in resistance to obesity [44]. Conversely, *PDK4* inhibits pyruvate oxidation and promotes fatty acid synthesis. *PDK4* knockout mice display reduced hepatic gluconeogenesis and lower blood glucose levels due to increased glucose utilization [45]. In skeletal muscle, *GLUT4* is an insulin-regulated glucose transporter that facilitates the uptake of glucose into fat and muscle cells [46]. Decreased expression of *GLUT4* in mice results in increased serum glucose and insulin levels, as well as reduced muscle glucose uptake [47]. Simultaneously, *NR1D1* can regulate *PPARGC1A* and promote mitochondrial biogenesis, resulting in increased replication of mitochondrial DNA and transcription of genes [48]. The expression of these genes is crucial for determining lipid accumulation in the host, which, in turn, corresponds to the accumulation of fatty acid material in the intestine. Additionally, *ALOX12B* encodes arachidonic acid 12-lipoxygenase, which acts on various polyunsaturated fatty acid substrates to produce biologically active lipid mediators [49]. *PLA2G2F*, an enzyme that hydrolyzes phospholipids into fatty acids and other lipophilic molecules, may play a role in the production of lipid mediators during inflammatory conditions [50]. The high level of arachidonic acid in the intestine of Dorper sheep may be attributed to the gene functions of *ALOX12B* and *PLA2G2F*.

Interactions between the gut-brain axis play a crucial role in regulating feeding behavior, growth, and intestinal motility. Melanocortin receptors play a crucial role in regulating appetite, food intake, and energy expenditure. The *MC4R* response to leptin signaling serves as a link between food intake and the regulation of body weight [51–53]. Research has demonstrated that a deficiency in *MC4R* leads to increased pulsatile secretion of growth hormone, resulting in significant increases in both human height and weight [54]. The NPY/AgRP nerve signaling pathway regulates melanocortin signaling by directly inhibiting melanocortin neurons and indirectly antagonizing the action of α-MSH on *MC4R*, thereby promoting feeding [55]. Our results indicate that Tan sheep are more active in feeding. Moreover, arachidonic acid serves as a precursor for prostaglandins, which play a crucial role in muscle contraction and neuromodulation [56]. Additionally, *ACHE* terminates neuronal transmission and signaling between synapses, which can influence the peristaltic contraction of the intestine [57]. These results suggest that Dorper sheep exhibit heightened intestinal signaling, which promotes intestinal peristalsis.

Although this study identified metabolic relationships between metabolites and genes, it is important to note that the metabolite database is incomplete, and there may be substances that have not been effectively annotated. Additionally, there may be metabolic relationships between immune-related genes and hormone receptor genes in growth and development that have not been deciphered and require further investigation [58]. Furthermore, in order to obtain a more comprehensive understanding of the relationship between environmental effects and metabolism, it is necessary to have larger sample sizes and utilize more advanced tools.

## Conclusion

In this study, we analyzed the role of gut metabolites and the combined regulatory networks of multiple tissues in the formation of breed-specific traits. Compared to the two breeds, Dorper sheep are known for their fast growth rate, while Tan sheep are known for their high fat content, particularly the high intermuscular fat content, which contributes to better meat quality. Dorper, a breed of sheep, increases purines and pyrimidines in a way that enhances amino acid synthesis. This promotes body development by improving glycine, serine, and threonine processes. The signal of cholesterol homeostasis is enhanced in Tan sheep muscle, which promotes lipid accumulation by up-regulating the *LDLR* and *LIPG* genes. This study presents an analytical case for interpreting the mechanisms of breed trait formation by conducting a comprehensive analysis of transcriptomes and metabolomes across multiple tissues.

## Materials and methods

### Experiment design and sample harvesting

A total of eight sheep, consisting of four ewes numbered 1–4 and four rams numbered 5–8 from each breed, were sourced from a breeding farm located in Shizuishan, Ningxia, China (38.59′00''N, 106.22′58''E). The sheep were raised under standard animal husbandry conditions, which included maintaining consistent temperature and humidity (with an annual average temperature of 8.4℃-9.9℃ and annual rainfall of 160–190 mm). They were also provided with the same feed, with lambs being fed ewe's milk and adults being fed mainly corn straw and alfalfa hay. The sheep had the same grazing time and access to an ample supply of drinking water. The diets provided were free of antibiotics, and the nutritional content met or exceeded the recommendations outlined in the NRC (2012).

At the age of 8 months (240 days), all sheep underwent a 24-h fast while being provided with access to drinking water before sacrifice. Subsequently, the sheep were weighed, anesthetized, and euthanized by jugular vein puncture [59]. Blood samples were collected from the anterior vena cava of each sheep using a procoagulant tube. These samples were then centrifuged at 3000 g for 15 min at 4 °C to separate the serum, which was subsequently stored in a freezer at -80 °C. Approximately 5 cm of midgut tissue was excised from the cecum and colon of all individuals, and the intestinal contents were extruded into lyophilization tubes. The intestinal tissues were washed in PBS twice, then sectioned and transferred into lyophilization tubes before being rapidly submerged in liquid nitrogen. As previous studies have found significant differences in ewes between breeds [18], we promptly collected the parietal region of the brain, the right lobe of the liver, and the triceps muscle from each ewe (a total of four ewes for each breed) and immediately froze them in liquid nitrogen. Finally, all tissue samples were preserved at -80 °C for further analysis.

### Histomorphology analysis

The liver tissues were fixed in paraformaldehyde and then subjected to freezing sectioning using a Leica frozen section machine (Leica Camera Inc., Wetzlar, Germany). Subsequently, the frozen slices were stained with oil red O and hematoxylin. They were then examined using the bright field of the ECHO imaging microscope (Discover Echo Inc., San Diego, CA, USA). Paraformaldehyde-fixed colons were dehydrated using graded ethanol and then embedded in paraffin. Sections of paraffin-embedded colon tissues, stained with hematoxylin and eosin (H&E), were examined under bright field illumination using an ECHO imaging microscope (Discover Echo Inc., San Diego, CA, USA) to assess variations in the thickness of the muscularis propria in the intestinal tissues. GraphPad Prism 8.0 was used for t-test statistical analysis, with significance indicated by *P-value < 0.05 and extreme significance indicated by **P- value < 0.01.

### Tissue RNA extraction, library preparation, and sequencing

We extracted total RNA using TRIzol (Invitrogen, Carlsbad, CA, USA) following the manufacturer's guidelines. RNA purity was quantified using a NanoDrop2000 spectrophotometer at 260 and 280 nm (Thermo Fisher Scientific, Waltham, MA, USA). The RNA integrity of the constructed library was determined using the Agilent 2100 Bioanalyzer (Agilent Technologies, Santa Clara, CA, USA) before performing paired-end sequencing on an Illumina HiSeqTM 2500 (Illumina BioTek Inc., San Diego, CA, USA). For sample labeling, we designated brain, liver, and skeletal muscle as B, L, and M, respectively. Additionally, D and T were used to denote Dorper and Tan sheep, respectively.

### Quality control and mapping of reads

After sequencing, the raw FASTQ data was obtained. Cutadapt was used to remove adapters located at the 3' end, and reads with an average quality score below Q20 were filtered out. The clean reads were aligned with the sheep reference genome (ARS-UI_Ramb_v2.0_genomic) using HISAT2 to obtain specific sequence feature information and location information on the reference genome or gene corresponding to the sequenced samples.

### Transcriptome bioinformatics analysis

To assess the quality of sequencing and the adequacy of sequencing data, we performed analyses on gene coverage uniformity and saturation. Gene expression levels were normalized using the FPKM method, which takes into account the effects of sequencing depth and gene length on reads. This enables a direct comparison of gene expression differences between two samples. The expression levels of various gene types in the genome annotation file were tallied using the results of the expression calculation. Differential expression analysis was performed using EdgeR between the groups, with | log_2_Foldchanges |> 1 and a *P-value* < 0.05 as the screening criteria. The Benjamini–Hochberg procedure was used as the default for multiple testing correction. Functional annotation of differentially expressed genes (DEGs) was carried out to identify GO and KEGG sets that were enriched with DEGs [60–62]. ClueGO was used to analyze the interrelationships of terms and functional groups in the biological network, facilitating the interpretation of biological functions of numerous genes and proteins [63]. A *P-value* of less than 0.05 was used as the cutoff value for pathway enrichment analysis.

### qRT-PCR Validation of Differentially Expressed Genes (DEGs)

*ABCB11* (ATP Binding Cassette Subfamily B Member 11), *NTCP* (Solute Carrier Family 10 Member 1), *PDK4* (Pyruvate Dehydrogenase Kinase 4), *PPARGC1A* (PPARG Coactivator 1 Alpha), *GLUT4* (Solute Carrier Family 2 Member 4), *ADIPOR2* (Adiponectin Receptor 2), *NPY* (Neuropeptide Y), *SST* (Somatostatin), *NR1D1* (Nuclear Receptor Subfamily 1 Group D Member 1), *β-actin* were selected for qRT-PCR analysis to verify the reliability and accuracy of transcriptomic data. The primers were designed using online National Center for Biotechnology Information (NCBI) Primer-BLAST tool (https://www.ncbi.nlm.nih.gov/tools/primer-blast/) and are shown in Table 1. Reverse transcription was carried out using the FastKing RT Kit (TaKaRa, Beijing, China), following to the manufacturer’s instructions. Quantitative Real-Time PCR (qRT-PCR) was carried out on BioRad CFX96 Real-Time PCR system (BioRad, United States), with SYBR® Green used as the fluorescent dye. With a total reaction volume of 20 μL, the mixture includes10 μL of 2 × SuperReal PreMix Plus (SYBR Green) (TIANGEN), 8 μL of RNase-free water, 0.5 μL of each forward and reverse primer (10 μM), and 1 μL of cDNA (approximately 200 ng). qRT-PCR amplification comprised an initial denaturation at 95 °C for 3 min, followed by 40 cycles of denaturation at 95 °C for 5 s, annealing and extension at 60 °C for 15 s, and as well as a melting/dissociation curve stage. *β-actin* served as an internal control to normalize the relative gene expression. Relative quantification of each gene included three technical replicates. The relative expression of genes was calculated using the 2 − ΔΔCt method [64]. All data was analyzed by one-way ANOVA. A *P-value* less than 0.05 was considered statistically significant.

**Table 1:** Primer information for quantitative PCR

RNA Name	Sequence of primer (5’-3’)
**ABCB11**	F: ACACCAACTACAGGAGTGCC
	R: CTTACTGCCGATGACCCTGG
**NTCP**	F: CCTCAATGTGACCTTCCCCC
	R: CAGAGCTGTTGGGAGGGTTT
**PDK4**	F: TGGTGTTCCCCTGAGAGTCA
	R: GTAACCAAAACCAGCCAGCG
**PPARGC1A**	F: GTGCAAGGGCAAACCACTTC
	R: GCTGTCATTTAGGGTGGCCT
**GLUT4**	F: CAGCTGCCTCCTACGAGATG
	R: CTAGCACCTGGGCGATTAGG
**ADIPOR2**	F: TCATTTTCTCTTCCGGCCCC
	R: CTGTGCTTTGTGGCTTCGAC
**NPY**	F: CTAGAAGAGCGCGCCAGAC
	R: TTGTCAGGCTTGGAGGGGTA
**SST**	F: ACCAGACAGAGAACGATGCC
	R: CATGGCGGGGTTTGAGTTAG
**NR1D1**	F: AGTGACAGCTCGAATGGCAG
	R: GGCGGAATGCTCCCAAAAGA
**Beta actin**	F: AGTACTCGGTGTGGATCGGA
	R: TCATGCAGCAAATGCTACGC

### Metabolome profile analysis

The cecum and colon metabolomes were separated by UHPLC and analyzed by mass spectrometry using a Q-Exactive high-resolution mass spectrometer (Thermo Fisher Scientific Inc., Waltham, USA). The raw mass spectrometry data underwent processing in Compound Discoverer 3.0 software (Thermo Fisher Scientific Inc., Waltham, USA) for peak extraction, alignment, correction, normalization, and other data preprocessing steps. This generated a 3D data matrix consisting of sample names, peak information (including retention times and molecular weights), and peak areas. Accurate mass matching (< 25 ppm) and secondary spectral matching were used to identify metabolite structures. Principal component analysis and sparse partial least squares discriminant analysis (sPLS-DA) were performed using MetaboAnalyst 5.0 (https://www.metaboanalyst.ca/) to create robust and easily interpretable models. A threshold of VIP > 1 and *P* < 0.05 was used to screen for differentially accumulated metabolites (DAMs). For the classification and KEGG (Kyoto Encyclopedia of Genes and Genomes) pathway enrichment analysis of metabolites, the MetOrigin platform was employed. It is accessible at http://metorigin.met-bioinformatics.cn/app/metorigin. The significance level used for pathway enrichment analysis was set at a *P-value* < 0.05.

### Correlation analysis of transcriptome and metabolome

To evaluate the impact of genetic factors on intestinal metabolite function, redundancy analysis (RDA) was conducted using the 'segRDA' package in R. *P-values* were obtained using a random permutation non-parametric test, with a significance level set at* P* < 0.05 to indicate a significant influence of genetic factors on metabolites. Correlations between intestinal metabolites and gene expression were estimated using Spearman correlation analysis with the 'Pheatmap' package in R (version 3.3.1). To comprehensively analyze the metabolic pathways involving genes and metabolites, we utilized MetaboAnalyst. The impact of these pathways was determined using the Signaling Pathway Impact Analysis (SPIA) package in R (version 3.3.1).

### Supplementary Information

**Additional file 1: Table S1.** Rawdata and map summary.

**Additional file 2: Table S2.** Statistical analysis of differentially expressed genes.

**Additional file 3: Table S3. **Enrichment analysis of differentially expressed genes.

**Additional file 4: Table S4.** Statistics of differentially accumulated metabolites in positive and negative ion mode.

**Additional file 5: Figure S1.** Multivariate statistical analysis of DAMs.

## Data Availability

All data and materials supporting our findings are included in the manuscript. The datasets generated and during the current study are available in the NCBI SRA repository, [PRJNA983762].
