# Supplementary figures and images for: Combined transcriptome and metabolome analysis reveals breed-specific regulatory mechanisms in Dorper and Tan sheep
Source: BMC Genomics. 2024 Jan 17;25:70. doi: 10.1186/s12864-023-09870-9 (PMC10795462; doi:10.1186/s12864-023-09870-9)

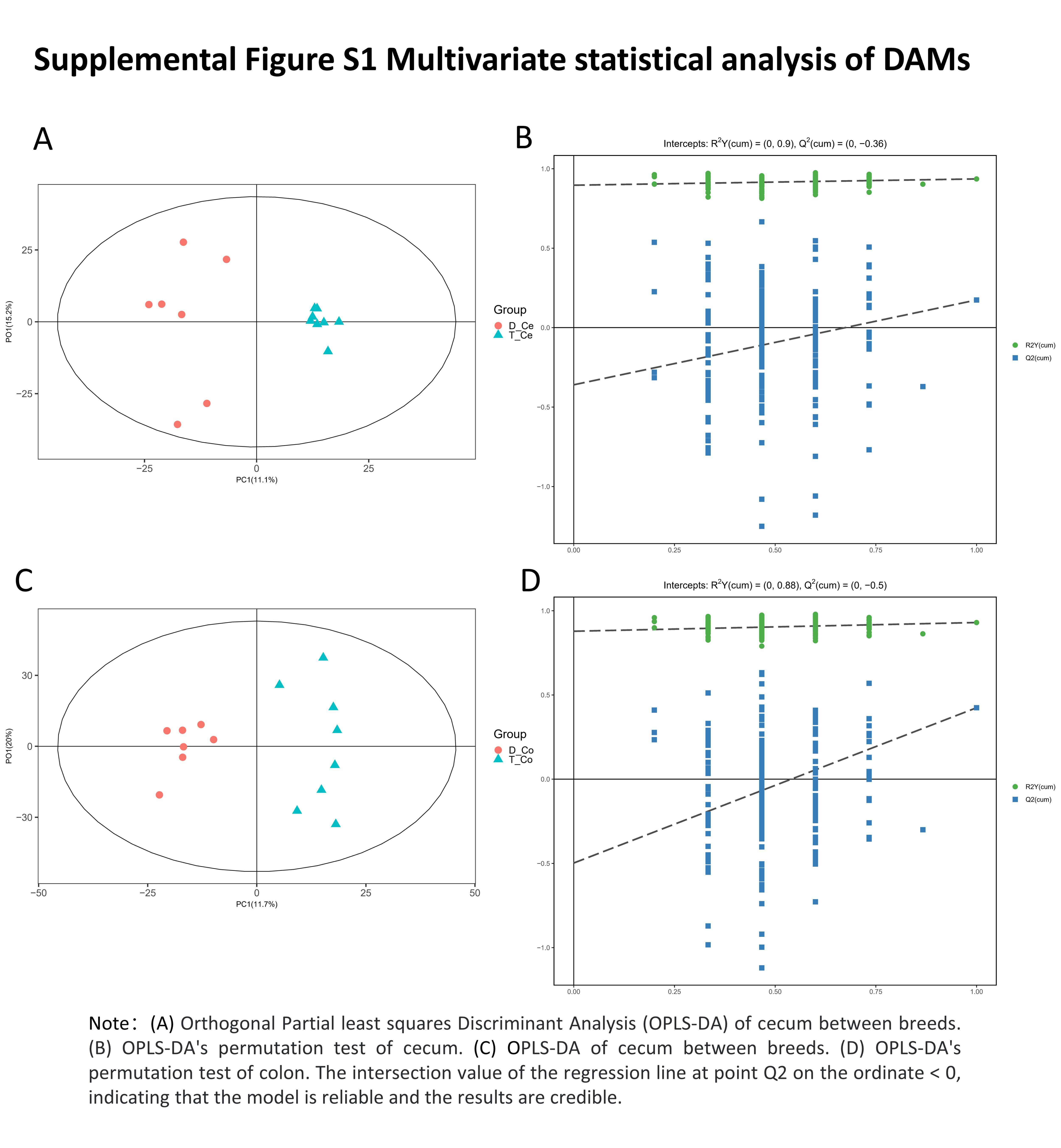

Supplement: Supplementary file 5 — Additional file 5: Figure S1. Multivariate statistical analysis of DAMs. [file 12864_2023_9870_MOESM5_ESM.jpg]
